# Wearable-Based Assessment of Cardiac Recovery After a Modified Bruce Test in Women with Breast Cancer: Role of Physical Activity and Treatment Duration

**DOI:** 10.3390/s26061996

**Published:** 2026-03-23

**Authors:** Carlos Navarro-Martínez, Natalia Ferrer-Artero, Keven Santamaria-Guzman, José Pino-Ortega

**Affiliations:** 1 Education, Diversity and Quality Research Group, Faculty of Education, University of Murcia, Campus de Espinardo, 30100 Murcia, Spain; c.navarromartinez@um.es; 2 BioVetMed & SportSci Research Group, Faculty of Sport Sciences, University of Murcia, 30720 San Javier, Spain; natalia.f.a@um.es; 3 Locomotor and Movement Control Laboratory, School of Kinesiology, Auburn University, Auburn, AL 36849, USA; kgs0071@auburn.edu

**Keywords:** wearable sensors, heart rate recovery, breast cancer, exercise testing, cardiovascular monitoring, modified Bruce protocol

## Abstract

**Highlights:**

**What are the main findings?**

- Wearable heart rate monitoring identified a statistically differentiated recovery interval at approximately 45–60 s, reflecting a progressive HR decline rather than a discrete physiological threshold, following a Modified Bruce test in women with breast cancer.
- Weekly physical activity hours and oncological treatment duration were significant predictors of heart rate recovery, whereas age showed no significant association.

**What are the implications of the main findings?**

- Wearable-based heart rate recovery assessment provides a feasible and practical tool for individualized cardiovascular monitoring in oncological exercise settings.
- Recovery intervals of around 45–60 s may be considered when designing personalized exercise prescriptions for women with breast cancer.

**Abstract:**

Heart rate recovery (HRR) is an important indicator of cardiovascular autonomic function, yet evidence in women with breast cancer remains limited. This study aimed to analyze heart rate recovery during the first two minutes following a maximal exercise test and to examine its association with age, weekly physical activity, and oncological treatment duration using wearable technology. A cross-sectional design was applied in 22 women with breast cancer enrolled in an oncological exercise program. Participants performed a maximal treadmill test using the Modified Bruce Protocol, after which the mean heart rate was recorded across eight 15 s recovery intervals using a wearable chest-strap heart rate sensor integrated with an inertial device (WIMU PRO). Results showed a progressive and significant decrease in heart rate during recovery, with the first statistically significant pairwise difference emerging at 45–60 s post-exercise compared to the initial recovery interval (*p* < 0.05), within the context of a continuous HR decline. Regression analysis identified weekly physical activity hours (β = −0.281, *p* = 0.013) and oncological treatment duration (β = −0.245, *p* = 0.038) as significant predictors of mean heart rate recovery, explaining 4.8% of the variance, while age was not significantly associated (β = 0.049, *p* = 0.622). In conclusion, a differentiated recovery pattern emerged at approximately 45–60 s post-exercise, with weekly physical activity and oncological treatment duration as determinants. These findings support the use of wearable-based monitoring to inform individualized exercise prescription in women with breast cancer.

## 1. Introduction

Advances in medicine and early detection have enabled increasing survival rates among women diagnosed with breast cancer in developed countries [1]. Despite these improvements in oncological treatment and prognosis, significant sequelae persist following surgery, radiotherapy, and chemotherapy, directly affecting survivors’ quality of life [2,3]. Among these adverse effects, treatment-related cardiac impairment stands out as one of the most relevant long-term complications, particularly in relation to chemotherapy-induced cardiotoxicity [4,5].

Physical exercise has been widely recognized as a non-pharmacological strategy to mitigate these adverse outcomes. Combined strength and aerobic training interventions have been shown to improve functional capacity, cardiorespiratory fitness, emotional well-being, and overall quality of life, while reducing cancer-related fatigue [6]. Furthermore, breast cancer survivors frequently present reduced physical activity levels and diminished functional performance compared to healthy counterparts, reinforcing the need for structured and individualized exercise programs in oncology populations [7]. In addition, improved cardiorespiratory fitness and habitual physical activity are associated with a lower risk of breast cancer incidence and recurrence, and exercise interventions in survivors have demonstrated consistent benefits in physical and functional outcomes [8,9,10].

Given the importance of cardiovascular health in this population, the identification of sensitive physiological markers capable of reflecting functional adaptation and cardiovascular risk is essential. In this context, heart rate recovery (HRR) after exercise has emerged as a clinically relevant indicator. Delayed HRR has been associated with increased cardiovascular risk and all-cause mortality [11], and it is widely considered a surrogate marker of autonomic nervous system function and prognosis [12]. Rapid recovery reflects efficient parasympathetic reactivation and adaptive cardiovascular regulation, whereas slower recovery has been linked to autonomic dysfunction and a higher likelihood of adverse cardiovascular events, such as arrhythmias or hypertension [13].

However, HRR should not be considered a single or uniform construct. Its interpretation depends on several methodological factors, including the exercise protocol employed, the intensity achieved during testing, the time frame selected for post-exercise assessment (e.g., 1 min, 2 min, or continuous monitoring approaches), and the analytical approach adopted (absolute heart rate values, relative changes from peak, or temporal slopes) [13]. Traditionally, HRR has been quantified using discrete time points, most commonly at 1 or 2 min post-exercise [14]. While clinically practical, this approach may oversimplify the dynamic nature of cardiovascular recovery, potentially overlooking relevant temporal patterns occurring within shorter intervals during the early recovery phase.

In this regard, wearable heart rate monitoring technology offers the opportunity to capture continuous physiological responses during and after exercise in a non-invasive and ecologically valid manner [15]. High-resolution temporal segmentation of recovery periods allows for a more detailed characterization of cardiovascular dynamics, extending beyond isolated measurement points and enabling functional assessment in applied oncology exercise settings.

Standardized treadmill protocols, such as the Bruce protocol and its modified version, are widely used to evaluate functional capacity and cardiovascular response in both clinical and research contexts [16,17,18]. Although these protocols are frequently employed to estimate aerobic capacity, the scientific literature examining detailed heart rate dynamics during recovery following maximal treadmill testing in women with breast cancer remains limited. Most HRR studies have been conducted in the general population or in individuals with established cardiovascular disease, leaving a gap in the oncological field [19].

Specifically, there is a lack of research addressing the fine-grained temporal characterization of heart rate during the early post-exercise phase in women with breast cancer using wearable monitoring systems. Moreover, the influence of factors such as age, physical activity level, and training experience on recovery dynamics in this population has not been sufficiently explored.

Therefore, the aim of this study was to characterize the heart rate pattern recovery during the first two minutes following a maximal exercise test performed under a modified Bruce protocol in women with breast cancer using wearable heart rate monitoring technology. Additionally, we sought to examine the association between recovery metrics and age, weekly physical activity time, and oncological treatment duration. We hypothesized that women with greater weekly physical activity and shorter treatment duration would exhibit more favorable recovery dynamics compared to those with lower activity levels or longer treatment exposure.

## 2. Materials and Methods

### 2.1. Study Design

This is a cross-sectional quantitative study aimed at analyzing post-exercise recovery in women who have survived breast cancer and are actively managing their condition, following the methodology of previous studies that have examined physical condition, and recovery in this population [19,20,21]. Given the cross-sectional and observational nature of this study, the associations explored between age, weekly physical activity, training experience, and heart rate recovery should be interpreted as correlational; therefore, causal inferences cannot be established. The Modified Bruce Protocol was implemented. This protocol is used for older adults or those with low physical capacity, allowing initial effort loads to be lower and increasing work and exercise duration [16]. To reduce potential sources of bias, all participants followed the same standardized exercise protocol, recovery procedure, and heart rate monitoring system, and were assessed under identical environmental and supervision conditions.

### 2.2. Participants

A total of 22 adult women with breast cancer participated in this study (age = 55 ± 7 years; height = 1.62 ± 0.07 m). All participants regularly attended the training sessions of the Chair of Exercise, Education, and Cancer at the University of Murcia. Given the rarity of the target population and the constraints of clinical recruitment, all eligible participants enrolled in the Oncology Exercise Program during the study period were included, constituting a total population sample. As such, a formal a priori sample size calculation was not applicable.

A purposive sampling strategy was used, as all participants were already enrolled in the Oncology Exercise Program and met the predefined inclusion and exclusion criteria. At the time of assessment, 50% of the sample was undergoing active oncological treatment, and 50% was in a disease-free phase (treatment-free interval). This clinical heterogeneity (active treatment vs. disease-free phase) may influence cardiovascular responses and heart rate patterns during recovery and was therefore considered when interpreting the findings. Participants’ demographic, clinical, and training characteristics are summarized in Table 1.

Inclusion criteria were: (i) adult women with a breast cancer diagnosis; (ii) enrollment in the Oncology Exercise Program; and (iii) medical clearance to perform physical exercise and the maximal treadmill test. Exclusion criteria were: (i) diagnosed severe cardiovascular disease or any medical contraindication to maximal exercise testing; (ii) advanced bone metastasis; or (iii) physical, musculoskeletal, or neuromuscular limitations that could compromise safety or prevent safe completion of the protocol. A total of 22 women met the eligibility criteria during the recruitment period, all of whom agreed to participate and completed the testing protocol. No participants were excluded after enrollment, and no missing outcome data were recorded. This approach prioritized participant safety and ethical considerations in this clinical population.

### 2.3. Instruments

**Figure 1:**
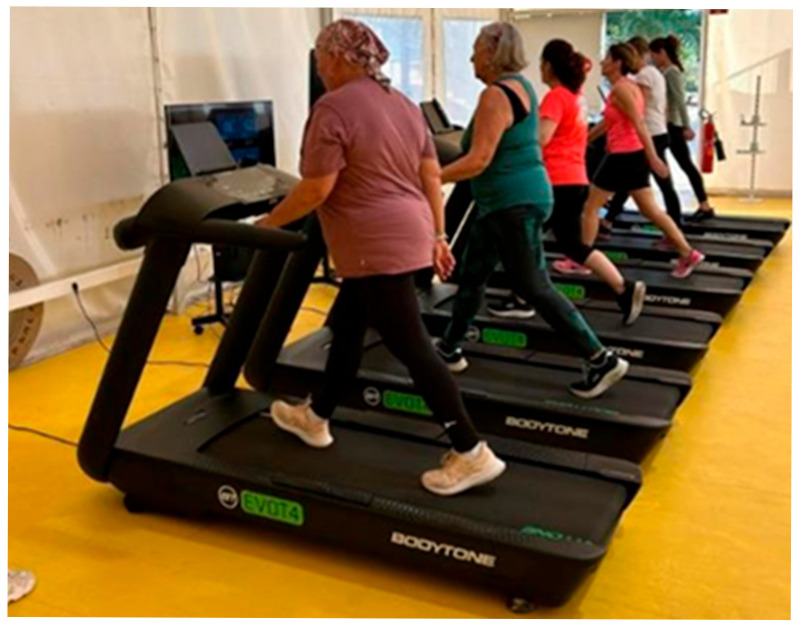
Participants performing the Modified Bruce Test on treadmills.

Treadmill equipment. A treadmill capable of increasing speed and platform height was used (EVOT4, Bodytone International Sport, Murcia, Spain) (Figure 1).

Heart rate monitoring and data acquisition. Each participant wore a chest-strap heart rate sensor (Coospo H6, Shenzhen Coospo Tech Co., Shenzhen, China) to monitor heart rate at a sampling frequency of 4 Hz. The HR sensor was linked to the WIMU PRO inertial device (RealTrack Systems, Almería, Spain), which received HR data wirelessly via the ANT+ protocol (low-latency transmission). During the test, participants visualized their HR in real time using WIMUFIT software (version 1.0.0.787, RealTrack Systems, Almería, Spain) displayed on a television placed in front of the treadmill. To ensure stable reception, the WIMU PRO receiver was positioned on the treadmill at a distance of <1 m from the chest strap, minimizing relevant signal losses.

Synchronization, signal quality control, and processing. To determine the exact onset of the test, a wireless device sent a synchronization marker to the WIMU PRO via ANT+. This time stamp was automatically stored and later used as the reference for data processing. After export, HR recordings were imported into SPro software (version 989, RealTrack Systems, Almería, Spain). A detailed visual inspection of the HR time series was performed to identify artifacts, potential dropouts, outliers, or discontinuities. When irregular segments were detected, the software’s automatic correction procedures based on interpolation were applied to ensure signal continuity and physiological plausibility. Based on the synchronization time stamp, the recovery period was segmented into consecutive 15 s intervals using the SPro segmentation tool, ensuring identical duration and consistent alignment across participants.

The WIMU PRO device has shown validity and reliability for HR quantification in previous studies [22,23]

Exercise test protocol. The Modified Bruce Protocol rubric [24] was used as the evaluation instrument. The Modified Bruce Protocol is a graded treadmill test in which speed, incline, or both increase every 3 min. Each stage is associated with estimated VO_2_max and METs, as shown in Table 2.

### 2.4. Procedure

Participants completed informed consent, which was approved by the Institutional Review Board of the University of Murcia (reference M10/2025/156). Prior to performing the Bruce Protocol, a brief and structured warm-up was conducted, consisting of general joint mobility exercises and progressive cardiovascular activation through dynamic movements such as heel-to-toe and jumping jacks, which increased body temperature and prepared the cardiovascular and neuromuscular systems [25]. Additionally, recent studies demonstrated that dynamic warm-ups before exercise tests can optimize initial physiological response without negatively affecting performance [26]. The pre-test warm-up was standardized for all participants and performed under the same conditions. Given that the recovery analysis was based on heart rate values relative to peak exercise and recorded immediately after test termination, the warm-up phase was not expected to differentially affect post-exercise heart rate during recovery comparisons across participants.

Familiarization with the treadmill began, asking participants to walk at a speed less than 0.75 m/s with 0% grade. After 3 min, stage I of the protocol started (Table 2).

The exercise test was terminated at volitional exhaustion, defined as the moment when the participant reported maximal perceived fatigue and was unable to continue maintaining the required workload despite standardized verbal encouragement from the evaluator. Immediately after test termination, participants initiated a standardized active cool-down on the treadmill at 1.38 m/s and 1% incline for 3 min to ensure a consistent post-exercise recovery condition across the sample. Heart rate during recovery was analyzed during the first 120 s immediately after test termination, corresponding to the initial 2 min of this standardized active cool-down period.

Weekly physical activity hours were obtained through self-report, based on the questionnaire completed by participants at the time of assessment. This variable reflected participants’ total habitual weekly physical activity and was used as an exploratory predictor in the regression model.

Data collection was conducted between February and March 2025.

### 2.5. Statistical Analysis

Data was analyzed using JASP 0.95.4. Descriptive statistics were presented as mean and standard deviation for each recovery interval. Data normality was assessed through visual inspection of Q-Q plots and the Shapiro–Wilk test. Homogeneity of variances was evaluated using Levene’s test.

To examine differences in mean heart rate across the eight recovery intervals, a one-way analysis of variance (ANOVA) was performed. Post hoc pairwise comparisons were performed using Bonferroni correction. Effect size was estimated using eta squared (η^2^). Although heart rate measurements were recorded repeatedly within each participant across the eight intervals, a one-way ANOVA was applied treating interval means as the unit of analysis, consistent with the exploratory and descriptive nature of this study. This approach was selected to characterize group-level temporal trends in heart rate during recovery rather than to model individual trajectories. The authors acknowledge that repeated-measures ANOVA or linear mixed-effects models would more rigorously account for within-subject dependence; these approaches are recommended for confirmatory analyses in future studies with larger samples.

Linear regression analysis was performed to examine the relationship between heart rate and predictor variables, including weekly hours of physical activity (self-reported through the questionnaire completed at the time of assessment), oncological treatment duration (in months), and age. Significance level was set at α = 0.05 for all statistical tests.

## 3. Results

The one-way ANOVA revealed significant differences in mean heart rate across the eight recovery intervals, F (7, 168) = 18.49, *p* < 0.001, η^2^ = 0.435, indicating a large effect size according to Cohen’s criteria. Levene’s test confirmed homogeneity of variances (F (7, 168) = 0.097, *p* = 0.998), supporting the use of standard ANOVA without correction.

### 3.1. Post Hoc Comparisons

Bonferroni-corrected post hoc comparisons revealed multiple significant differences between recovery intervals. The first statistically significant difference was observed between interval 1 (0–15 s) and interval 4 (46–60 s; mean difference = 15.62 bpm, 95% CI [3.97, 27.26], *p* = 0.002, Cohen’s d = 1.241), indicating that, within the context of a continuous HR decline, a statistically differentiated reduction was first detectable at 45–60 s post-exercise. The most relevant findings regarding exercise prescription are in Table 3:

These results demonstrate that, in our sample, from 45 to 60 s post-exercise (beginning of interval 4), HR was significantly lower than during the initial post-exercise intervals, indicating a differentiated temporal pattern of heart rate recovery dynamics rather than a discrete physiological threshold.

**Figure 2:**
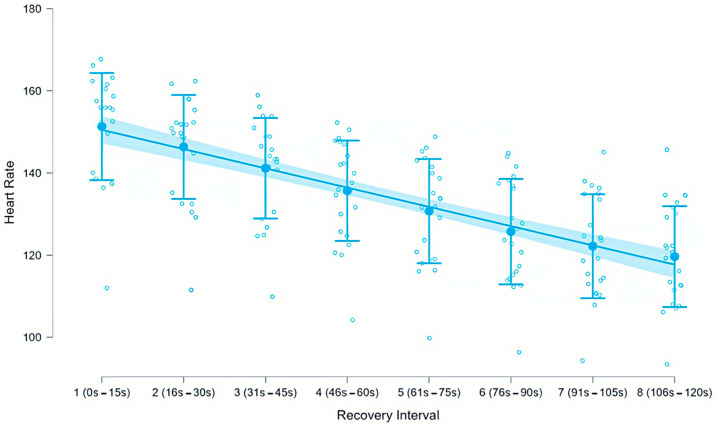
Mean heart rate (bpm) across eight consecutive 15 s recovery intervals following the Modified Bruce test in women with breast cancer (*n* = 22). Filled circles represent interval means, error bars indicate standard deviations, and open circles represent individual participant values. The solid line depicts the linear trend of heart rate decline across the recovery period, with the shaded band representing the 95% confidence interval of the fitted regression line. Heart rate decreased progressively from interval 1 (0–15 s; ~151 bpm) to interval 8 (106–120 s; ~120 bpm), reflecting a continuous and significant reduction throughout the two-minute post-exercise recovery phase.

Figure 2 provides a graphical representation of heart rate changes across the eight 15 s recovery intervals.

### 3.2. Multiple Linear Regression Analysis

A linear regression model including three predictors (weekly physical activity hours, oncological treatment duration in months, and age) was evaluated to examine associations with mean heart rate during recovery. The model was statistically significant (F (3, 164) = 2.734, *p* = 0.045, R^2^ = 0.048, adjusted R^2^ = 0.030). No influential cases were detected (Cook’s distance analysis). Descriptive statistics for mean heart rate across the eight recovery intervals are presented in Table 3. Mean heart rate decreased progressively from 151.3 ± 13.0 bpm (interval 1; 0–15 s) to 119.7 ± 12.3 bpm (interval 8; 106–120 s), representing a total mean reduction of approximately 31.6 bpm over the two-minute recovery period.

Weekly physical activity hours (β = −0.281, t = −2.508, *p* = 0.013, 95% CI [−0.497, −0.065]) and oncological treatment duration (β = −0.245, t = −2.088, *p* = 0.038, 95% CI [−0.738, −0.021]) were identified as significant negative predictors of mean HR during recovery. Age was not a significant predictor in this model (β = 0.049, t = 0.494, *p* = 0.622, 95% CI [−0.337, 0.559]).

## 4. Discussion

This study examined heart rate during recovery during 120 s divided into eight 15 s intervals, as well as demographic and behavioral factors that could influence this cardiovascular response in women with breast cancer. Results revealed two main findings: (1) a statistically differentiated temporal pattern of recovery was observed: from 45 to 60 s post-exercise onward, mean HR was significantly lower than during the earliest recovery interval. This finding reflects a progressive, continuous HR decline in which a statistically discernible difference first emerged at that interval, and should not be interpreted as a discrete physiological threshold; (2) weekly physical activity hours and oncological treatment duration were significant predictors of heart rate during recovery, explaining 4.8% of the variance, while age did not contribute significantly to mean heart rate during recovery.

Heart rate recovery (HRR) has been previously recognized as an indicator of cardiac autonomic function and cardiovascular recovery capacity [27]. Our initial hypothesis proposed that women with greater weekly physical activity and shorter treatment duration would exhibit more favorable recovery dynamics. Results of this study support this hypothesis: the regression model identified weekly physical activity hours and treatment duration as significant predictors, suggesting that, in the present sample, women with higher habitual physical activity and shorter treatment exposure exhibited lower mean heart rate during early recovery after exercise. Contrary to expectations, age did not show a significant association with mean heart rate during recovery in this oncological population.

Although heart rate recovery (HRR) is traditionally defined as the difference between peak heart rate and heart rate at fixed time points such as 1 or 2 min post-exercise, the present study aimed to characterize the early recovery phase using a higher temporal resolution. By analyzing heart rate in 15 s intervals and considering mean heart rate during recovery, we sought to capture the dynamic profile of cardiac recovery rather than relying on a single discrete time point. This approach may provide a more comprehensive representation of recovery kinetics, particularly in clinical populations where recovery patterns can be heterogeneous.

The significant association between weekly physical activity hours and lower mean HR during recovery aligns with the broader literature recognizing habitual physical activity as a modifiable factor that enhances cardiovascular efficiency and promotes parasympathetic reactivation. The significant inverse association between treatment duration and mean HR during recovery may reflect cumulative physiological adaptation over the survivorship continuum, or alternatively, greater cumulative exposure to supervised exercise among participants with longer program participation. However, given the cross-sectional design, causal inferences cannot be established, and these interpretations should be considered hypothetical. The modest explained variance (R^2^ = 0.048) indicates that additional clinical variables not assessed in the present study—such as cardiotoxicity biomarkers, medication use, and autonomic function markers—likely contribute substantially to variability in heart rate during recovery variability in this population.

Our results align with those found in studies such as Groarke et al. [28], which compared healthy women with women with breast cancer, showing that this special population presents significantly higher prevalence of abnormal HRR at minute 1 (clinically defined as a decrease of ≤ 12 beats/min). Although those studies relied on conventional fixed-point HRR metrics, our study focused on the continuous early recovery profile through 15 s interval monitoring, complementing the literature by identifying a specific temporal pattern where, in our sample, recovery occurs at approximately 45–60 s, which could provide additional information for exercise prescription.

Other studies supporting part of our results regarding the effect of supervised exercise are Giallauria et al. [29], which demonstrated that regular supervised physical exercise practice improves HRR in women with breast cancer over 12 months, going from 18 to 23 beats/min average decrease, while a sedentary control group showed no significant changes. Similarly, Knobf et al. [30] found that, in cancer-surviving women, a combined intervention of aerobic and resistance training (similar to the training applied to participants in this study) for one year resulted in a significant improvement in HRR at minute 1 after an exercise test compared to a control group that only received home-based physical activity recommendations. These longitudinal findings are consistent with our cross-sectional results showing that greater weekly physical activity hours were associated with lower mean HR during recovery, providing convergent evidence that habitual physical activity positively influences cardiac recovery capacity in women with breast cancer.

To contextualize our findings, it is necessary to contrast them with available evidence in the general population and individuals with cardiovascular disease. In healthy subjects, a rapid heart rate drop after exercise is a sign of good vagal tone and physical condition. Classically, a reduction of ≤12 beats/min at recovery minute (in active recovery protocol) has been defined as deficient, as it indicates insufficient parasympathetic reactivation [28]. Even other studies associated that a decrease of less than 42 beats at two minutes after a submaximal exercise test entails ~2.5 times greater risk of death over a 12-year period [17]. This highlights that, in individuals without evident disease, slow cardiac recovery is a potent prognostic factor for mortality.

Identification of the recovery pattern around 45–60 s in our sample has potential practical implications. Our findings may be interpreted in the context that women with breast cancer present specific clinical and pathophysiological characteristics that could influence cardiovascular responses differently from the general population. In general terms, post-exercise heart rate recovery is commonly described as reflecting an initial phase dominated by parasympathetic reactivation during the first 30–60 s, followed by a slower phase in which sympathetic withdrawal may contribute to further reductions in heart rate. However, since autonomic function was not directly assessed in the present study (e.g., HRV or other autonomic markers), these mechanistic interpretations should be considered plausible explanatory frameworks rather than definitive conclusions. In this context, our results indicate that heart rate values became significantly lower from approximately 45–60 s onward compared with the earliest recovery intervals, supporting a differentiated temporal pattern of early recovery in this sample.

In our study, women with greater weekly physical activity showed lower mean heart rate during recovery, consistent with the well-established cardioprotective effects of regular exercise on autonomic function and cardiac efficiency [31,32,33]. The significant association with treatment duration may reflect differential adaptation pathways over the survivorship continuum, warranting further investigation in longitudinal designs. Additionally, it must be considered that breast cancer-surviving women present greater autonomic imbalance after adjuvant treatments, showing resting heart rates 9–16% higher than healthy women of the same age [29].

The absence of a significant age effect on mean HR during recovery in the present study contrasts with findings from the general population, where age is frequently identified as a key determinant of HRR [27]. This may suggest that in this specific oncological population, the cardiotoxic burden of cancer treatments—including the effects of anthracyclines, trastuzumab, and hormonal inhibitors on the myocardium, autonomic conduction system, and vascular endothelium [34,35,36]—may attenuate or override the typical age-related differences in cardiac recovery. Within a sample where all participants are exposed to these treatment-related cardiovascular alterations, modifiable behavioral factors such as habitual physical activity may emerge as more discriminating predictors of recovery capacity. However, this mechanistic interpretation remains speculative, as cardiotoxicity and autonomic markers were not directly assessed in the present study.

This study presents several limitations that must be considered when interpreting results. First, the relatively small sample size (*n* = 22) limits statistical power to detect more subtle associations and generalizability of findings. This sample size also limits our ability to perform subgroup analyses or explore more complex interactions between variables. In addition, participants were recruited from a single Oncology Exercise Program, which may introduce selection bias and limit external validity. Therefore, findings may not be fully generalizable to women with breast cancer who are sedentary or not engaged in structured rehabilitation/exercise programs. The absence of a control group with healthy individuals to contrast results and establish specific normative values for the oncological population is considered. Second, the cross-sectional design does not allow establishing causal relationships or evaluating longitudinal changes in heart rate during recovery, limiting our ability to determine whether observed differences are cause or consequence of training status or age. Third, given the repeated interval-based heart rate measurements within each participant, the use of simple ANOVA rather than repeated-measures ANOVA or linear mixed-effects models represents a methodological limitation inherent to the exploratory scope of this study. Future confirmatory analyses should employ hierarchical modeling approaches to more rigorously account for within-subject dependence across recovery intervals. Therefore, interval-level inferences should be interpreted with caution and confirmed in future studies using hierarchical modeling approaches.

Fourth, several potentially relevant confounding factors were not systematically recorded or controlled. These include cardioactive medications, hormonal and emotional state, baseline cardiovascular comorbidities, and detailed treatment-related variables such as chemotherapy type and cumulative dose, radiotherapy field (including potential cardiac exposure), use of known cardiotoxic agents (e.g., anthracyclines), and time elapsed since treatment completion. In addition, objective cardiotoxicity and cardiac function measures (e.g., biomarkers such as troponins or natriuretic peptides, or ventricular function assessed by echocardiography), as well as anthropometric and body composition indicators (e.g., BMI, fat mass, and lean mass), were not available for inclusion in the models. Moreover, exercise-related factors such as recent training adherence, exercise intensity, or physical activity performed in the 24 h prior to testing were not standardized, which may have influenced heart rate during recovery variability. Finally, clinical heterogeneity in treatment status (50% in active treatment and 50% in a disease-free phase) may have introduced additional variability in cardiovascular responses. Given the modest sample size, exploratory subgroup comparisons according to treatment phase were not performed, and this should be considered when interpreting the findings.

It is recommended that future studies consider including a control group of healthy individuals to contrast results, account for individual variability, and take into account factors such as medications, hormonal and emotional states that can also influence cardiac recovery, as well as perform longitudinal interventions.

Results should not be extrapolated to sedentary breast cancer survivors or patients with severe cardiovascular comorbidities without further investigation.

## 5. Conclusions

The main study findings demonstrate that, in the participants evaluated, heart rate during recovery showed significant changes at 45–60 s post-exercise, when mean HR differed significantly from its initial state immediately after effort cessation. In women with breast cancer participating in a structured oncology exercise program, this statistically differentiated interval observed in our sample may offer a preliminary reference point for structuring rest periods of approximately 45–60 s between intense exercise sets, pending confirmation in larger controlled studies, although confirmation in larger studies with controlled experimental designs is required.

Weekly physical activity hours and oncological treatment duration emerged as significant predictors of heart rate during recovery, with greater physical activity and shorter treatment duration associated with lower mean HR during recovery. Contrary to the initial hypothesis, age was not a significant predictor of heart rate during recovery in this cross-sectional oncological sample. This may be related to cardiovascular changes associated with cancer treatment but requires confirmation in future studies including cardiotoxicity indicators. However, these findings should be interpreted cautiously in light of the cross-sectional design, the clinical heterogeneity of the sample (active treatment vs. disease-free phase), and the limited statistical power associated with the modest sample size.

Given the relatively small sample size, cross-sectional design, and the limited control of potential confounding variables, the present findings should be interpreted as exploratory. Future research should include longitudinal follow-up designs to examine changes in heart rate during recovery across different treatment phases and investigate the differential effects of specific exercise modalities (e.g., aerobic versus resistance training) on post-exercise cardiac recovery in women with breast cancer.

## Figures and Tables

**Table 1:** Demographic, clinical, and training characteristics of the participants (*n* = 22).

Category	Variable	Value
Demographic	Age (years), mean ± SD	55 ± 7
	Height (m), mean ± SD	1.62 ± 0.07
Clinical status	Clinical phase: Active treatment	50%
	Clinical phase: Disease-free phase (treatment-free interval)	50%
Treatment type (current or past)	Chemotherapy (received or ongoing)	55%
	Surgery only	15%
	Radiotherapy only	15%
	Chemotherapy + radiotherapy	10%
	Surgery + radiotherapy	5%
Lymphedema	No	85%
	Yes	15%
Training/physical activity	Training experience: 1–4 years	55%
	Weekly physical activity (4–6 h/week)	36%

**Table 2:** Description of speed and incline stages in the Bruce Protocol.

Step	Time (min)	Speed (m/s)	Incline %	VO_2_max mL/kg/min	Mets
I	3	0.75	0	14–18	5
II	6	0.75	5	14–18	5
III	9	0.75	10	14–18	5
IV	12	1.11	12	23–25	6–7
V	15	1.50	14	28–34	8–10
VI	18	1.86	16	35–40	11–12
VII	21	2.44	18	46–50	13–15
VIII	24	2.50	20	51–59	14–17
IX	27	2.66	22	60–65	18–20

Speed values are expressed in meters per second (m/s) for consistency with the measurement system, although the Bruce protocol is traditionally reported in mph or km/h.

**Table 3:** Significant Post Hoc Comparisons—Bonferroni-Corrected Pairwise Comparisons.

Recovery Intervals		Mean Difference	t	Cohen’s d	*p*_bonf_
1	4	15.616	4.116	1.241	0.002
	5	20.589	5.427	1.636	0.001
	6	25.587	6.745	2.034	0.001
	7	29.12	7.676	2.314	0.001
	8	31.662	8.346	2.516	0.001
2	5	15.636	4.122	1.243	0.002
	6	20.634	5.439	1.64	0.001
	7	24.167	6.371	1.921	0.001
	8	26.709	7.04	2.123	0.001
3	6	15.44	4.07	1.227	0.002
	7	18.974	5.001	1.508	0.001
	8	21.515	5.671	1.71	0.001
4	7	13.505	3.56	1.073	0.014
	8	16.046	4.23	1.275	0.0001

Only statistically significant pairwise comparisons are shown (*p* < 0.05).

## Data Availability

The data presented in this study are available from the corresponding author upon reasonable request, due to ethical and privacy considerations related to the study population.

## Abbreviations

The following abbreviations are used in this manuscript:

ANOVA	Analysis of Variance
HR	Heart Rate
HRR	Heart Rate Recovery
PA	Physical Activity
METs	Metabolic Equivalents
WIMU	Wearable Inertial Measurement Unit
